# Case report: Insulinoma masquerades as epilepsy – quantitative EEG analysis

**DOI:** 10.3389/fneur.2024.1371055

**Published:** 2024-03-26

**Authors:** Natalia Kostolanska, Petr Klimes, Jitka Kocvarova, Hana Pikulova, Ondrej Strycek, Milan Brazdil, Irena Dolezalova

**Affiliations:** ^1^Faculty of Medicine, Masaryk University, Brno, Czechia; ^2^Institute of Scientific Instruments of the CAS, Brno, Czechia; ^3^First Department of Neurology, St. Anne’s University Hospital Brno, Masaryk University, Member of the ERN EpiCARE, Brno, Czechia; ^4^Department of Neurosurgery, University Hospital Brno, Brno, Czechia

**Keywords:** hypoglycemia, insulinoma, acute symptomatic seizures, epilepsy, EEG postprocessing

## Abstract

Insulinomas are rare gastrointestinal tumors with an incidence of 1–3 per million inhabitants annually. These tumors result in excessive insulin production, culminating in hypoglycemia. Such hypoglycemia triggers various central nervous system (CNS) manifestations, including headache, confusion, abnormal behavior, and epileptic seizures, which can lead to misdiagnosis as epilepsy. This case report documents a 46-year-old male who presented seizure-like episodes. Episodes occurred mainly during the night, lasting several minutes to hours. Initial seizures were characterized by bizarre behavior and altered responsiveness. Over time, seizure frequency, complexity, and severity escalated. We managed to record two episodes during long-term EEG and report, as the first ones, the detailed quantitative EEG analysis of these hypoglycemia-related events. EEG changes preceded the development of clear-cut pathological motor activity in tens of minutes and were present in all investigated frequency bands. The development of profound motor activity was associated with other increases in EEG power spectra in all frequencies except for delta. The most pronounced changes were found over the left temporal region, which can be the most susceptible to hypoglycemia. In our patient, the seizure-like episodes completely disappeared after the insulinoma removal, which demonstrates their relationship to hypoglycemia.

## Introduction

Insulinomas are rare gastrointestinal tract tumors with an annual incidence of 1–3 cases per million inhabitants (1). Sporadic incidence predominates (2). Insulinomas are characterized by excessive insulin production, leading to hypoglycemia. Hypoglycemia leads to central nervous system (CNS) manifestations, including headache, blurred vision, confusion, dizziness, abnormal behavior, amnesia, and tonic–clonic seizures (3). Less typically, the neurological presentation can encompass focal neurological deficits or focal seizures.

Seizures occurring alongside hypoglycemia can be classified as acute symptomatic seizures. These seizures occur in close temporal proximity to an acute central nervous system insult, which could be metabolic, toxic, structural, infectious, or due to inflammation. Metabolic disturbances encompass alterations in serum glucose levels and sodium, magnesium, calcium, creatinine, and urea nitrogen. Both low (<2 mmol/L or < 36 mg/dL) and high (> 25 mmol/L or > 450 mg/dL) serum glucose levels are associated with seizures (4). Although patients with hypoglycemia typically manifest bilateral tonic–clonic seizures, a considerable portion of reports describe seizures characterized by peculiar movements or behavior (5).

This article is the first report focusing on quantitative EEG analysis during seizure-like episodes in insulinoma.

## Case report

We present a case report of a 46-year-old man who started to experience seizure-like episodes. These episodes were predominantly linked to nighttime, lasting tens of minutes.

**Figure 1:**
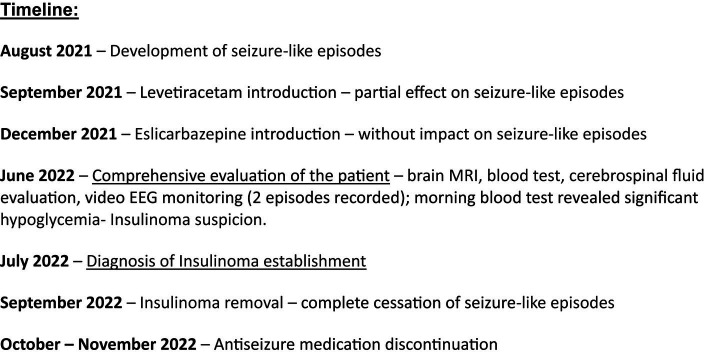
The timeline.

At first, the seizures appeared in the early morning hours and were described as episodes of bizarre behavior, including grunting, blowing, sitting up, or rocking movements. The patient was mainly unresponsive to verbal stimuli. An inadequate reaction was present if partial responsiveness was perceived. Both seizure frequency and severity progressed over time. The frequency increased to 4 to 5 seizures per month and the duration to 3 h. The timeline of disease development is summarized in Figure 1.

Antiseizure medication (ASM, levetiracetam) was introduced with a temporary effect. However, increasing doses and a second drug (eslicarbazepine) influenced neither seizure frequency nor severity. The patient underwent a comprehensive evaluation (blood test, cerebrospinal fluid examination, the examination of antibodies against paraneoplastic and autoimmune encephalitis, MRI, and scalp EEG); blood tests, including blood glucose level, were normal. The blood glucose level was measured when no clinical symptoms were present. Interictal EEG showed intermittent slow activity above the left temporal region, but no interictal epileptiform discharges were current.

**Figure 2:**
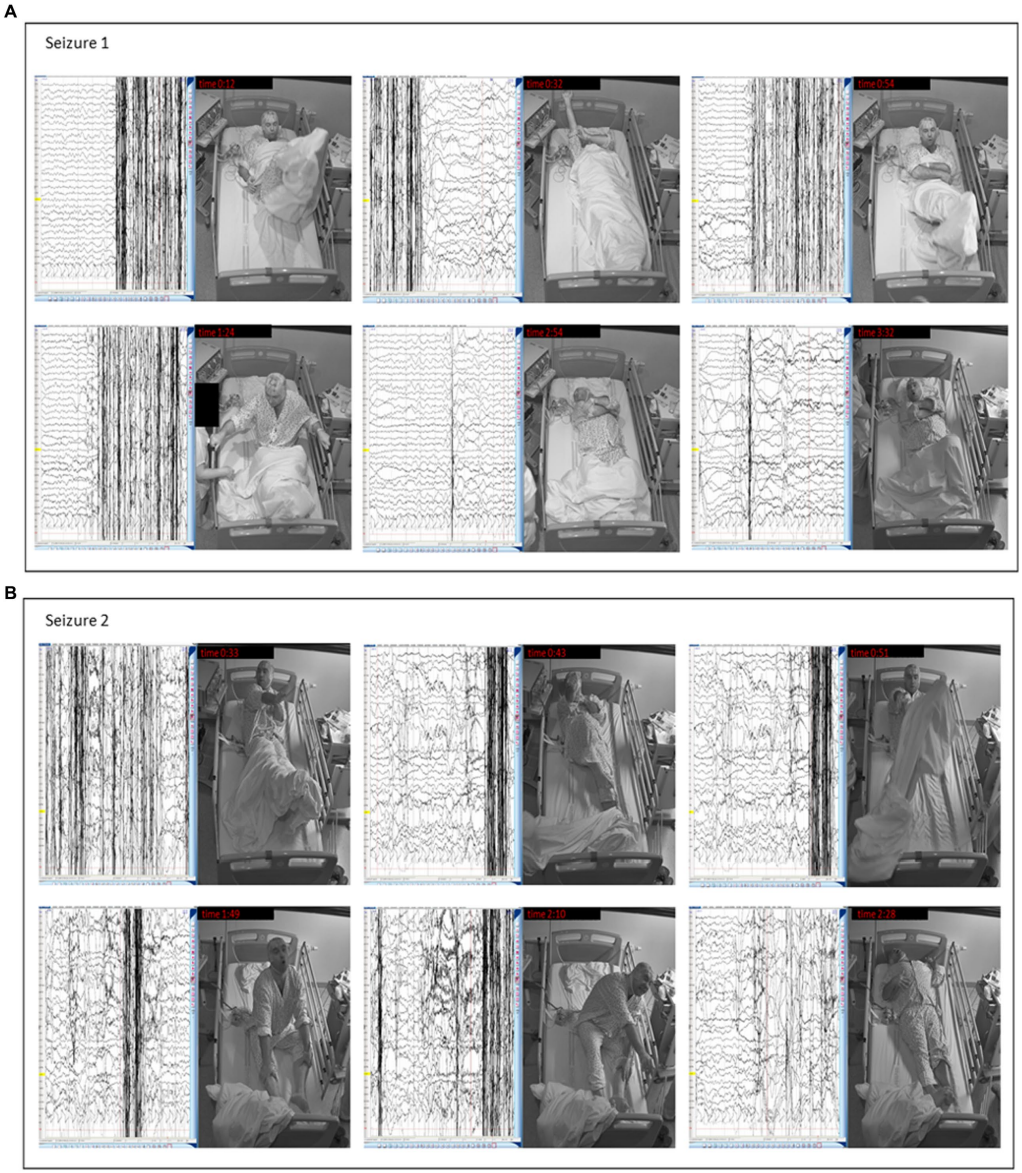
Clinical manifestation of insulinoma in our patient – seizures captured during video EEG. **(A)** Seizure 1 – Video-EEG: The seizure starts with rocking movements (0:12). At 0:32, the patient raises his right hand, throws his legs on the bed, and straightens his shirt sleeve. Rocking motions are repeated (0:54). After the nurse arrives, he says he wants to sleep. He counts aloud at the nurse’s request but only to 2. Then he rocks again, sits up, and grabs the bed rails (1:24). He crosses his arms (2:54) and grunts. At the end of the seizure, he raises his head and hyperventilates (3:32). **(B)** Seizure 2 – Video-EEG: The seizure again begins with rocking movements. The patient raises his right hand and lifts his legs (0:33). He then kicks his feet from side to side and flexes his right leg (0:43). He rocks again (0:51), sits down, and makes loud noises, tossing his head up and down. He does not recognize the apple and says it is a hat (1:30). At 1:49. He rocks violently, squeals, flails his hands, and stops responding. The seizure ends with arm-stretching (2:10) and tense body posture (2:28).

Two typical episodes were captured during video EEG monitoring (Figures 2A,B). Subsequently, routine morning blood tests showed a blood glucose level of 0.5 mmol/l (9 mg/dL), which led to the establishment of a correct diagnosis of insulinoma.

Thirteen months after the first symptoms, the patient underwent surgical removal of the insulinoma. Histological diagnosis confirmed pancreatic insulinoma pT1, N0, M0, and G2. After surgery, the patient was seizure-free, and antiseizure medication was withdrawn.

## EEG analysis

We divided the EEG recording before and during the episodes into two segments: pre-ictal state and ictal state. When visually analyzing the pre-ictal state, there was slow activity predominantly from the theta range with occasional delta over both temporal regions. This activity lasted tens of minutes (90 min in the first seizure and 60 min in the second). It could be misinterpreted as sleep, but no rest electroencephalographic events (K-complexes, sleep spindles, or vertex waves) were recorded. Moreover, the patient had opened his eyes and performed some inconspicuously occasional activities (lip smacking, blinking, infrequent hand movements) but was able to respond. None of those mentioned activities were evaluated as abnormal. Suddenly, a massive motor manifestation developed.

In the presented case report, we focused on EEG signal processing and quantitative analysis. We divided EEG into 3 segments: (1) Rest – waking state before seizure-like episode development (patient lying on the bed with opened eyes), (2) Pre-ictal segment – characterized by the predominance of slow activity as described above, but no profound seizure manifestation is present, and (3) Ictal segment– characterized by clear-cut pathological motor activity.

**Figure 3:**
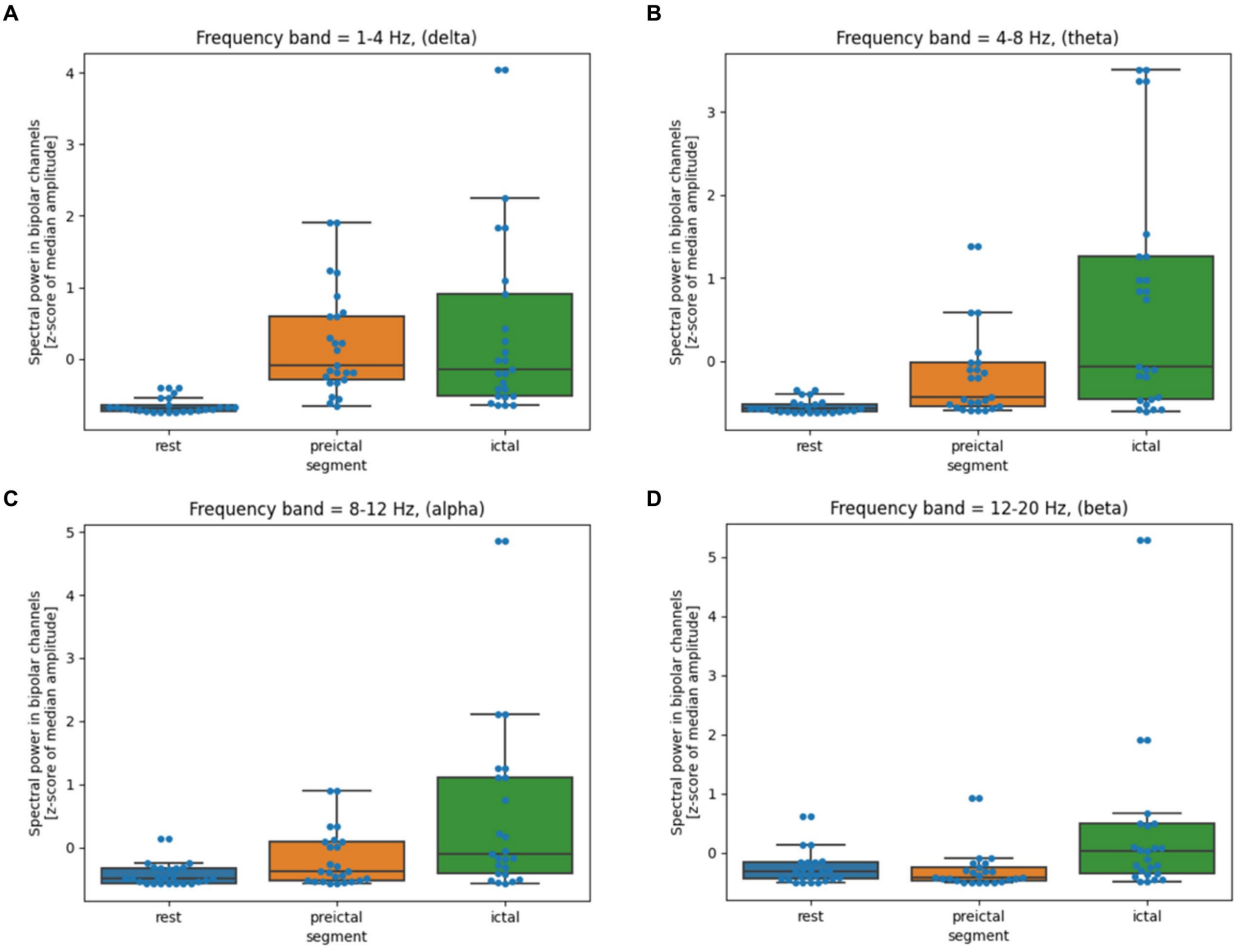
**(A–D)** Quantitative EEG analysis during hypoglycemic episodes. The box shows the quartiles of the dataset, while the whiskers extend to show the rest of the distribution. The blue dots are the individual data points. The middle line shows the median.

The analyzed segments of 2 h of rest, 33 min of pre-ictal, and 108 min of ictal were visually inspected for artifacts. Any visible artifacts were removed from further analysis. The recordings were analyzed in the standard bipolar montage. Spectral power was calculated using an overlapping 1-s window in the following frequency bands: 1–4 Hz, 4–8 Hz, 8–12 Hz, and 12–20 Hz. The median value for each segment and each bipolar channel was calculated. Non-parametric, paired Wilcoxon signed-rank test with Bonferroni correction for multiple comparisons found statistically significant differences in all frequency bands, even between the Rest and Pre-ictal segment (all *p*-values<0.01). Moreover, there were statistically significant changes between Pre-ictal and Ictal segments in all frequencies (all *p*-values<0.01) except for delta (*p*-value = 1). Two pairs of electrodes (el. T3-T5 and T3-Sz1) demonstrated the highest degree of change (Figure 3).

## Discussion

Insulinomas are rare pancreatic neuroendocrine neoplasms that can be misdiagnosed as epilepsy (6). In a recent comprehensive review by Dudley et al. (7), the authors identified 20 studies containing 22 case reports in which hypoglycemic episodes resembled epilepsy. The staggering fact is that out of 22 patients, 19 were misdiagnosed, and the time between the onset of the disease and the establishment of the correct diagnosis ranged from 2 weeks to 8 years, with a median of 10 months (7). Insulinomas accounted for the most common cause of hypoglycemia in 20 out of 22 patients.

Seizures occurring alongside hypoglycemia can be classified as acute symptomatic seizures. Hypoglycemia triggers acute symptomatic seizures through intricate metabolic changes that are not yet fully understood, but the imbalance of neurotransmitters in the extracellular space is pivotal (4). In essence, glucose is metabolized intracellularly to provide energy for cellular function through glycolytic reactions (Embden-Meyerhof-Parnas pathway) and the subsequent Krebs cycle. Glucose metabolism leads to acetate production. Acetate combines with oxaloacetate to form citrate, a primary molecule in the Krebs cycle. In simplified terms, the lack of glucose leads to an insufficiency of acetate, resulting in an inadequate reaction with oxaloacetate and its accumulation. The accumulation of oxaloacetate accelerates a reaction that generates aspartate, an excitatory neurotransmitter. Aspartate, flooding into the extracellular space, elevates its concentration by 1,600%. Another metabolic pathway also heightens the concentration of the inhibitory neurotransmitter gamma-aminobutyric acid (GABA), albeit to a lesser extent, rendering the imbalance more skewed toward excitation. These processes chiefly underlie acute symptomatic seizures and subsequent brain damage (4).

The lack of glucose as a pivotal source of energy for brain functioning has a long-lasting impact on cerebral functioning, as could be supported by quantitative EEG analysis. In the pre-ictal period lasting tens of minutes, we recorded diffuse slowing, distinguishable from normal wakefulness, sleep, and ictal period. Clinically, the patient was even fully responsive in a pre-ictal state, but no detailed testing was performed. That is why we cannot exclude a milder degree of mental status alternation. When quantitative EEG analysis was completed, all frequency bands showed statistically significant differences between rest and preictal periods. Based on this finding, we can expect that brain activity is altered in the presence of hypoglycemia in the long period before the clear seizure manifestation. Another clear-cut change in EEG spectral power was present when the motor manifestation started. We recorded the statistically significant difference in all frequency bands except delta. It is possible that the level of brain glucose metabolism reached a critical value, provoking more global brain dysfunction, which led to the motor manifestation and the patient’s unresponsiveness. These changes were most pronounced over the left temporal region, which could be susceptible. It is questionable whether temporal lobes represent universally sensitive areas or whether this susceptibility was present only in our patient.

The proper diagnosis of insulinoma can be encouraged by Whipple’s triad consisting of (1) symptoms consistent with hypoglycemia, (2) plasma glucose <2.8 mmol/L (50.4 mg/dL) measured during the symptomatic phase, and (3) symptom relief upon increasing plasma glucose concentration (8).

In our patient, several misleading factors were identified. Firstly, our patient reported no subjective issues other than seizure-like episodes. Hypoglycemia-related problems were not reported.

Secondly, he reported, a fact confirmed by his wife, a transient reduction in seizure frequency and severity following the administration of ASM. Two possible explanations exist. ASM might have functioned as a placebo. Another possibility is that nighttime hypoglycemia was partially suppressed by increased glucose intake in the evening hours. In retrospect, when we inquired, the patient admitted to consuming a small sweet drink before sleep after dinner, coinciding with the introduction of ASM.

Thirdly, another misleading factor was the EEG characteristics. We noted focal slow abnormalities in the interictal state over the left temporal region. Focal slow activity or interictal epileptiform discharges are relatively common in patients with this diagnosis. Only 32% of patients exhibited a normal EEG in Dudley et al. (7) review. In their study, ictal recordings were available for only 6 patients, all exhibiting diffuse slowing. In one case, this slow activity was more pronounced over posterior regions (5, 7).

In our case, the seizure semiology raised significant concern. All seizures occurred at night, likely due to nocturnal fasting, and were deemed atypical, marked by waxing and waning symptoms. High variability between individual seizures was noted, and the seizures were prolonged. These unusual features led to suspicion of non-epileptic seizures originating from somatic factors, prompting further investigation of our patient.

In our case, the critical examination that heightened suspicion of insulinoma was blood testing, which revealed severe morning hypoglycemia. This fact underscores the necessity of conducting blood tests (blood count, electrolytes [natrium, potassium, chlorides, calcium, magnesium, and phosphate], kidney and hepatic functions, C-reactive protein [CRP]) or other investigations (e.g., ECG or brain-imaging) during episodes of unclear origin to exclude acute symptomatic seizures or other possible causes (8).

The acute treatment of acute symptomatic seizures is concerned with correcting underlying conditions. This claim must be highlighted, especially in cases of acute symptomatic seizures caused by metabolic disturbance. When focusing on the management of hypoglycemia, the recommendations are as follows. The critical blood glucose value is 3.9 mmol/L (70 mg/dL). The management is dependent on whether the consciousness is preserved or not. In patients with preserved consciousness, the immediate administration of oral fast-acting carbohydrates is recommended (glucose tablets, sucrose, fructose, orange juice, Mentos, and jellybeans; there is no significant difference in the patient’s outcome). In patients with alert consciousness with no intravenous access (typical out-of-hospital setting), the administration of glucagon (subcutaneous or intramuscular) is recommended. Glucagon is a pancreatic hormone counterregulatory to insulin, i.e., raising blood glucose levels. In patients with alert consciousness and intravenous access (in-hospital setting), the administration of 10–50% dextrose is recommended. A starting bolus dose of 12.5–15 g of dextrose is recommended. If the hypoglycemia persists, a higher dose of 25 g dextrose should be administered. Some patients, especially individuals treated with basal insulin or sulfonylurea, require continuous infusion with dextrose (9).

In conclusion, the EEG changes associated with hypoglycemia in insulinomas have unique characteristics. The pathological slow activity is long-lasting and can be recorded several minutes before the onset of clear motor manifestation. The development of motor symptoms is associated with other changes in EEG spectra in all frequency bands.

## Patient perspective

The patient reports complete relief after the removal of the insulinoma. The patient especially appreciates that he is now entirely without any nighttime attacks and any ASM.

## Data availability statement

The datasets presented in this article are not readily available because of ethical and privacy restrictions. Requests to access the datasets should be directed to the corresponding author.

## Ethics statement

Ethical review and approval was not required for the study on human participants in accordance with the local legislation and institutional requirements. Written informed consent from the patients/participants or patients/participants’ legal guardian/next of kin was not required to participate in this study in accordance with the national legislation and the institutional requirements. Written informed consent was obtained from the individual(s) for the publication of any potentially identifiable images or data included in this article.

## Author contributions

NK: Conceptualization, Investigation, Visualization, Writing – original draft, Writing – review & editing. PK: Data curation, Formal analysis, Software, Visualization, Writing – review & editing. JK: Conceptualization, Data curation, Project administration, Writing – original draft. HP: Formal analysis, Investigation, Resources, Writing – review & editing. OS: Formal analysis, Investigation, Methodology, Software, Writing – review & editing. MB: Data curation, Methodology, Supervision, Validation, Visualization, Writing – review & editing. ID: Conceptualization, Investigation, Supervision, Validation, Writing – original draft, Writing – review & editing.
